# Molecular Typing and Antimicrobial Susceptibility of Methicillin-Resistant* Staphylococcus aureus* Isolated from Bovine Milk in Tanzania

**DOI:** 10.1155/2018/4287431

**Published:** 2018-03-12

**Authors:** Jibril Mohammed, Michael Henry Ziwa, Yaovi Mahuton Gildas Hounmanou, Adela Kisanga, Huruma Nelwike Tuntufye

**Affiliations:** ^1^Department of Microbiology, Parasitology and Biotechnology, College of Veterinary Medicine and Biomedical Sciences, Sokoine University of Agriculture, Morogoro, Tanzania; ^2^Department of Veterinary Medicine and Public Health, College of Veterinary Medicine and Biomedical Sciences, Sokoine University of Agriculture, Morogoro, Tanzania; ^3^Department of Veterinary and Animal Sciences, Faculty of Health and Medical Sciences, University of Copenhagen, 1870 Frederiksberg, Denmark

## Abstract

Methicillin-resistant* Staphylococcus aureus* (MRSA) in raw milk can be transmitted from animals to humans, and in Tanzania raw milk is sold in local markets and consumed as purchased. This study was performed to determine the molecular characteristics and antimicrobial susceptibility pattern of MRSA strains isolated from raw bovine milk sold at local markets in Tanzania. A total of 117 raw milk samples were cultured on Baird-Parker medium to isolate* S. aureus* and PCR was used for amplification of* gltB* gene for* S. aureus* identification and the presence of* mecA* gene for methicillin-resistant strains. Coagulase-negative (CN)* S. aureus* were reconfirmed using tube coagulase, DNase, and API Staph tests. MRSA isolates were* spa *typed whereas antimicrobial susceptibility testing was performed by the disc diffusion method. Forty-six coagulase positives (CP) and two CN* S. aureus* were identified. Most strains were resistant to penicillin (72%), and 3 isolates: 2 CN* S. aureus* and 1 coagulase-negative Staphylococci (CNS), were phenotypically resistant to vancomycin, oxacillin, and cefoxitin and were confirmed to carry* mecA. *Resistance to clindamycin, trimethoprim-sulfamethoxazole, and tetracycline was 23.9%, 30.4%, and 41.3%, respectively. Twelve isolates exhibited multidrug resistance; however, only one* mecA *positive strain among the three was typeable and belonged to* spa *type t2603. This study reports for the first time the presence of CN variant of MRSA, which was assigned the spa type t2603, and the presence of multidrug resistant* S. aureus* isolates from bovine milk in Morogoro, Tanzania.

## 1. Introduction

*Staphylococcus aureus* is an important opportunistic pathogen both in humans and in dairy cattle. It is also a common cause of mastitis in dairy cows [1], a primary reason for antibiotic use in dairy farms. The use of antimicrobial agents in dairy farms as well as in other food animal production systems is a major concern in the emergence of resistant zoonotic bacterial pathogens [2, 3]. Methicillin-resistant* Staphylococcus aureus *(MRSA) has emerged as a major cause of health care-associated (HA) and community-associated (CA) infections [4]. In addition to that, infection and colonization by MRSA have been well documented in several animal species and mostly caused by livestock-associated MRSA strains [5] and are frequently multidrug resistant (MDR). This can result in higher costs, longer treatment time, and higher rates of hospitalization and comorbidities [6].

The presence of MRSA in bovine milk and dairy environments poses potential risk to farm workers, veterinarians, and farm animals that are exposed to infected cattle. This study was conducted in Morogoro, which is one of the leading regions in livestock keeping in Tanzania, to investigate the occurrence of MRSA in milk samples. In Morogoro, milk collected from dairy farms is distributed to various local sales' points and markets, from which samples were taken for investigation.

## 2. Methods

### 2.1. Study Area and Study Design

This study was carried out between January and June 2015 in Morogoro Municipality, which had a total population of 316,603 persons [7]. The current study was a cross-sectional design that involved 18 of the 29 Wards. In each of the selected Wards, sales points and local shops where raw milk is sold were randomly selected. A total of 117 milk samples (4 to 8 samples from each Ward), of at least 10 ml each, were collected in labeled sterile Universal Bottles and transported with ice in sterile cool box to the laboratory for immediate processing.

### 2.2. Isolation and Identification of* Staphylococcus* Species

The fresh milk samples were cultured on Baird-Parker media (OXOID, Hampshire, England) at 37°C for 24 h and the presumptive isolates were subcultured for another 24 h followed by biochemical identification using tube coagulase and catalase tests. Coagulase-negative* S. aureus* were reconfirmed using tube coagulase, DNase, and API Staph tests with* S. aureus *ATCC 25923 and* Staphylococcus epidermidis *ATCC 12228 as positive and negative controls, respectively.

### 2.3. DNA Extraction

DNA was extracted using the boiling method as described by [11] with some modifications. Briefly, three to five bacterial colonies were added to 1.5 ml Eppendorf tubes containing 200 *μ*l of nuclease-free water. The tubes were boiled in water bath at 99°C for 10 min. After centrifugation at 30,000 ×g for 1 min, 3 *μ*l of supernatant was used as template in a 20 *μ*l PCR mixture.

### 2.4. PCR Detection of* S. aureus*

The detection of* S. aureus* was performed using primers (Macrogen Inc., Seoul, South Korea) which were species specific for* S. aureus *(Table 1). The PCR mixture contained an aliquot of 3 *μ*l bacterial DNA template, primers, and distilled water to a total volume of 20 *μ*l into AccuPower® PCR PreMix tubes (Bioneer Inco., South Korea). The mix contained 1 U* Taq* DNA polymerase and 250 *μ*M each of dNTP, 10 mM Tris-HCL (pH 9.0), 30 mM KCl, 1.5 mM MgCl_2_, stabilizer, and tracking dye. Each primer concentration was 0.4 *μ*M derived from a chromosomal DNA specific for* S. aureus* amplifying 108 bp product, which codes for the enzyme glutamate synthase* (gltB)*. The PCR mixtures were incubated in a TAKARA PCR Thermal Cycler Dice Gradient TP600 (Takara Bio, Tokyo, Japan). PCR conditions were as described by [9] with initial denaturation step at 95°C for 5 mins, and 35 cycles of amplification at 95°C for 30 sec, with annealing at 55°C for 30 sec, extension at 72°C for 30 sec, and final extension at 72°C for 5 min and a hold at 4°C.

### 2.5. PCR Detection of* mecA* Gene

The detection of* mecA* gene was carried out as a single target PCR amplification using the primer pairs listed in Table 1. All* S. aureus* and CNS isolates were screened for* mecA* gene for the genotypic identification of MRSA. The primer and PCR conditions were obtained from [8] with some modifications. The initial primer concentration was 0.046 *μ*M and amplicon size was 147 bp. The PCR was run in 20 *μ*l of AccuPower PCR PreMix tubes (Bioneer Inco., South Korea) containing 3 *μ*l template DNA, with cycling parameters beginning with an initial denaturation step at 95°C for 5 min followed by 35 cycles of 95°C for 30 sec, 52°C for 45 sec, and 72°C for 30 sec, ending with a final extension step at 72°C for 7 min and a hold at 4°C.

### 2.6. Spa Typing of* mecA* Carrying Isolates

For spa gene PCR, primer pair in Table 1 used for typing were derived from [10]. The PCR mixture contained 20 *μ*l of AccuPower PCR PreMix with 3 *μ*l bacterial DNA and concentration of 1 *μ*l each of the primers with variable product size (bp). PCR conditions were 94°C for 3 min; 35 cycles each of 94°C for 30 sec, 50°C for 30 sec, and 72°C for 60 sec; and a final extension at 72°C for 5 min. PCR products were purified using GeneJET purification kit (Thermo scientific). Samples were sequenced with the same primers used in PCR. Sequencing reactions used BigDye v3.1 sequencing mix (Applied Biosystems) and were cycled using 30 cycles of 96°C for 10 sec, 50°C for 5 sec, and 60°C for 2 min. Products were purified and separated on an ABI 3730 DNA Analyzer (Applied Biosystems). Chromatograms were analyzed using Ridom StaphType v2.2.1 software (Ridom GmbH).

### 2.7. Antimicrobial Susceptibility Test

The Kirby-Bauer Disk Diffusion Susceptibility test was used to obtain the antimicrobial resistance profile of the isolates for clindamycin (2 *μ*g), vancomycin (30 *μ*g), trimethoprim-sulfamethoxazole (25 *μ*g), tetracycline (30 *μ*), penicillin G (10 IU), oxacillin (1 *μ*G), and cefoxitin (30 *μ*g). Isolates were considered to be resistant to methicillin if they were resistant to both oxacillin and cefoxitin, with particular emphasis to cefoxitin which is a better inducer of* mecA* gene. Moreover, cefoxitin disk diffusion tests give clearer endpoints and are easier to read than oxacillin [12].* S. aureus* ATCC 29213 was used as reference strain and interpretation was done according to standard guidelines of Clinical and Laboratory Standards Institute [13].

## 3. Results

### 3.1. Prevalence of* S. aureus* in Raw Milk Samples

**Figure 1:**
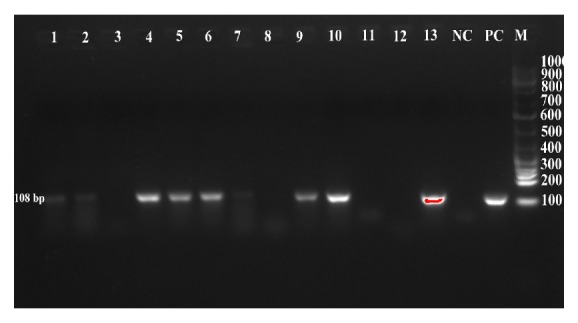
PCR detection for* S. aureus* isolates: lanes 3, 8, 11, and 12 are negative, while lanes 1, 2, 4, 5, 6, 7, 9, 10, and 13 are positive for* S. aureus* target gene* (gltB)* at 108 bp. NC and PC are negative and positive controls, respectively; M: DNA ladder marking from 100 bp to 1 kb.

A total of 117 raw milk samples were analyzed, of which 75 (64%) yielded coagulase-positive staphylococci (CPS) and 42 (36%) coagulase-negative staphylococci (CNS) presumptive isolates. PCR detected 46* S. aureus* (Figure 1) among CPS and 2* S. aureus *among the CNS, giving a 41% prevalence of* S. aureus* in raw milk in the Morogoro Municipality (Table 2). The two CN* S. aureus* were reconfirmed with tube coagulase, DNAse, and API Staph tests.

### 3.2. Antimicrobial Susceptibility Test

**Figure 2:**
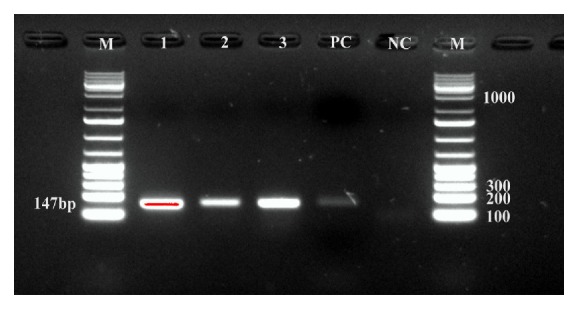
*PCR detection of mecA gene in coagulase-negative S. aureus and coagulase-negative staphylococci isolates: *PCR products showing* mecA* gene at 147-bp. Lane 1: coagulase-negative Staphylococci* mecA* positive; lanes 2 and 3: coagulase-negative* S. aureus mecA *positive; PC: positive control; NC: negative control; M: DNA marker.

The overall resistance to clindamycin, vancomycin, trimethoprim-sulfamethoxazole, tetracycline, penicillin G, oxacillin, and cefoxitin was 23.9%, 2.2%, 30.4%, 41.3%, 71.7%, 6.5%, and 4.4%, respectively. Resistance to both oxacillin and cefoxitin was seen in three isolates: 2 CN* S. aureus* and 1 CNS (Table 3) and they were confirmed to carry* mecA* (Figure 2).

Twelve (26.1%) of the CP* S. aureus* isolates exhibited multidrug resistance (MDR), whereas none of the CNS isolates were MDR (Table 4).

### 3.3. *Staphylococcus* Protein A (spa) Typing

Among the 3 isolates containing* mecA*, 1 CN* S. aureus* contained a* spa *gene and the primers produced a band of 1150 bp based on the repeat pattern spa type t2603; however, the other 2* mecA* positive isolates were untypable.

## 4. Discussion

This study found the prevalence of* S. aureus* in raw milk to be 41.0%, with samples from Msamvu (16.7%) and Mwembesongo (12.5%) being the most contaminated while those from Tungi (2.1%), Magadu (2.1%), Kihonda (2.1%), and Boma (2.1%) were the least contaminated. This frequency of contamination is similar to that reported in studies conducted in Morocco, Brazil, Ethiopia, and Kenya, which found frequencies of 40%, 68%, 48.7%, and 30.6%, respectively [14–17].

The milk samples were collected from sale points and open markets; therefore, the high frequency of contamination could be related to poor hygiene practices in handling milk at various stages from farms to the markets. It is also possible that the health status of the animals may have contributed to the occurrence of some of the isolates recovered, as previous studies have associated this with mastitis and other animal infections [18, 19]. However, this study did not investigate the health status of the dairy cows.

A significant variability was noted in susceptibility of* S. aureus *to the tested antibiotics, with lowest resistance to oxacillin (6.5%) and cefoxitin (4.4%), whereas the highest resistance was to penicillin (71.7%). Isolates that were methicillin-resistant, hence resistant to both oxacillin and cefoxitin, were 2 (4.4%)* S. aureus* and 1 (2.4%) CNS. These variations are most probably related to frequency of use, which is associated with cost and availability. Compared to CNS,* S. aureus* had slightly higher levels of resistance for all five classes of antibiotics tested. In Morogoro the most frequently used antibiotics in the livestock industry are oxytetracycline and sulphur-based antibiotics [20, 21]. These drugs are cheap and available over the counter and are frequently used inappropriately. The high level of resistance to the tested antibiotics has also been reported in human population residing in areas where the study was conducted [22], prompting suggestion of potential transmission of antimicrobial resistance genes between bacteria found in humans and animals. In Morogoro, a number of factors compound the problem of antimicrobial resistance in zoonotic infections. These include (i) tendency for animal owners to stock drugs in their houses and engaging unskilled people such as farmers/peasants themselves and animal attendants to treat animals [23] and (ii) high degree of drugs abuse/misuse by livestock keepers through failure in observing the recommended therapeutic doses and arbitrary drug combinations and nonobservance of withdraw periods [24]. Others include lack of basic knowledge of the concept of antibiotic resistance among livestock keepers [21].

In the present study PCR was conducted to detect* mecA* gene in all isolates. In CP* S. aureus* it was found that none of the isolates contained the gene while 1 CNS and 2 CN* S. aureus* harboured it. This study revealed the prevalence of CN-MRSA and MRCNS to be 4.2% and 2.4%, respectively. This finding is in accordance with previous studies conducted elsewhere, 4.0% [6], 4.8% [25], and 0 to 7.4% [26]. Identification of MRSA in milk in this study emphasizes the need for increased public awareness regarding safe food handling to help prevent cross-contamination [27] and urges for public health interventions, including decreasing the use of antibiotics [28, 29]. The 2 CN-MRSA showed resistance to only one class of antimicrobials although* mecA* gene is believed to confer resistance to most currently available beta-lactam antibiotics [30]. However, not all* mecA* positive clones are resistant to methicillin, and overall resistance level in a population of MRSA depends on efficient production of PBP-2a, which is modulated by a variety of chromosomal and extrachromosomal factors [31]. This explains why MRSA resistance levels range from phenotypically susceptible to highly resistant [30]. According to Tavares [32], the resistance to antibiotics is determined by not only the presence of resistance genes, but also the expression of these genes. The* S. aureus* may be pathogenic or nonpathogenic with the pathogenic strains usually exhibiting coagulase-positivity and causing disease in their hosts [33].

In conclusion, the prevalence of CN-MRSA and MRCNS in bovine milk was found to be 4.2% and 2.4%, respectively. The study reports for the first time the presence of presumptive coagulase-negative variant of MRSA and MRCNS in raw milk in Morogoro, Tanzania. Among the three* mecA* positive isolates, hence, only one of the coagulase-negative variants of MRSA was typeable and was assigned the spa type t2603. Based on the results of the current study, it is important to characterize further the CN-MRSA and MRCNS isolates to determine whether the isolates were clonally related. Furthermore, future studies for MDR* S. aureus *strains should be screened for detection of the novel* mecA* homologue* mecC* gene and other antibiotic resistance genes such as* mecB*. Further studies are also required to identify the origin of these MRSA strains to figure out whether they originated from milk sellers (during handling) or the animals as livestock-associated MRSA (e.g., from mastitis).

## Figures and Tables

**Table 1:** Summary of primers used in this study.

Primer	Sequence (5′-3′)	Amplicon size (bp)	Specificity	Reference(s)
MecA147-F MecA147-R	GTG AAG ATA TAC CAA GTG ATT ATG CGC TAT AGA TTG AAA GGA	147	*mecA*	[8]
Sa442-1 Sa442-2	AAT CTT TGT CGG TAC ACG ATA TTC TTC ACG CGT AAT GAG ATT TCA GTA GAT AATACA ACA	108	Species-specific Target	[9]
1095-F 1517 -R	AGA CGA TCC TTC GGT GAG C GCT TTT GCA ATG TCA TTT ACT G	Variable	*Spa*	[10]

**Table 2:** The *S. aureus* isolated from milk in the Morogoro Municipality.

Wards (Codes)	Samples analyzed	Positive samples	% of positive sample
Boma (1)	7	1	2.1
Mazimbu (2)	8	3	6.3
Mwebesongo (3)	8	6	12.5
Msamvu (4)	8	8	16.7
Kihonda (5)	8	1	2.1
Kichangani (6)	4	2	4.2
Kilakala (7)	6	4	8.3
Mafiga (8)	6	2	4.2
Kiwanjacha Ndege (9)	8	3	6.3
Sabasaba (10)	5	3	6.3
Chamwino (11)	7	3	6.3
Mafisa (12)	6	3	6.3
Mbuyuni (14)	5	2	4.2
Mji Mpya (15)	6	2	4.2
Kingolwira (16)	4	1	2.1
Tungi (17)	9	1	2.1
Mkundi (18)	4	2	4.2
Magadu (19)	8	1	2.1

Total number of *S. aureus* isolated from Municipality (*n* = 48) and the proportion of isolates from each Ward.

**Table 3:** Phenotypic and genotypic identification of methicillin-resistant isolates.

Isolates	Phenotype		Genotype
	OX/1 *µ*g	FOX/30 *µ*g	*mecA* gene
*S. aureus*	6.52% (*n* = 3)	4.35% (*n* = 2)	*n* = 2
CNS	19.05% (*n* = 8)	2.38% (*n* = 1)	*n* = 1

**Table 4:** Multidrug resistance pattern of *S. aureus* and CNS isolated from raw milk.

Number of antibiotic agents	*S. aureus* isolates (*N* = 46)		CNS isolates (*N* = 42)	
	Number	Percent	Number	Percent
0	9	19.6%	33	78.6%
1	12	26.1%	5	4.8%
2	13	28.3%	4	11.9%
3	9	19.6%	0	0%
4	2	4.4%	0	0%
5	1	2.2%	0	0%

## Abbreviations

CLSI: Clinical Laboratory Standards Institute
CN-MRSA: Coagulase-negative MRSA
CNS: Coagulase-negative staphylococci
DNA: Deoxyribonucleic acid
MRCNS: Methicillin-resistant coagulase-negative staphylococci
MRSA: Methicillin-resistant* Staphylococcus aureus*
PBP-2a: Penicillin-binding protein 2a
PCR: Polymerase chain reaction
SUA: Sokoine University of Agriculture.
